# Genetic Variability of Chrysodeixis Includens Nucleopolyhedrovirus (ChinNPV) and the Insecticidal Characteristics of Selected Genotypic Variants

**DOI:** 10.3390/v11070581

**Published:** 2019-06-26

**Authors:** Eduardo Aguirre, Inés Beperet, Trevor Williams, Primitivo Caballero

**Affiliations:** 1 Institute for Multidisciplinary Research in Applied Biology, Universidad Pública de Navarra, 31006 Pamplona, Navarra, Spain; 2 Departamento de Investigación y Desarrollo, Bioinsectis SL, Pol. Ind. Mocholi Plaza Cein 5, Nave A14, 31110 Noain, Navarra, Spain; 3 Instituto de Ecología AC, Xalapa 91073, Veracruz, Mexico

**Keywords:** genetic variants, plaque assay, bioassay, pathogenicity, speed-of-kill, bioinsecticide

## Abstract

Genetic variation in baculoviruses is recognized as a key factor, not only due to the influence of such variation on pathogen transmission and virulence traits, but also because genetic variants can form the basis for novel biological insecticides. In this study, we examined the genetic variability of Chrysodeixis includens nucleopolyhedrovirus (ChinNPV) present in field isolates obtained from virus-killed larvae. Different ChinNPV strains were identified by restriction endonuclease analysis, from which genetic variants were isolated by plaque assay. Biological characterization studies were based on pathogenicity, median time to death (MTD), and viral occlusion body (OB) production (OBs/larva). Nine different isolates were obtained from eleven virus-killed larvae collected from fields of soybean in Mexico. An equimolar mixture of these isolates, named ChinNPV-Mex1, showed good insecticidal properties and yielded 23 genetic variants by plaque assay, one of which (ChinNPV-R) caused the highest mortality in second instars of *C. includens*. Five of these variants were selected: ChinNPV-F, ChinNPV-J, ChinNPV-K, ChinNPV-R, and ChinNPV-V. No differences in median time to death were found between them, while ChinNPV-F, ChinNPV-K, ChinNPV-R and ChinNPV-V were more productive than ChinNPV-J and the original mixture of field isolates ChinNPV-Mex1. These results demonstrate the high variability present in natural populations of this virus and support the use of these new genetic variants as promising active substances for baculovirus-based bioinsecticides.

## 1. Introduction

Genetic variability within baculoviruses is strongly related to host-pathogen interactions within each insect during viral infection. The genetic structure of baculovirus populations arises from the need to productively infect genetically diverse host insects and maximize the production of progeny viruses in the host to improve the likelihood of subsequent transmission [1,2]. This variation in the genetic variants that conform baculovirus isolates is generated by multiple mechanisms, including point mutation, insertion and deletion, recombination and horizontal gene transfer. As a consequence, baculovirus isolates comprise mixtures of genetic variants. This allows variants to cooperate and complete among themselves, in interactions that modulate the survival of these viruses in nature [3,4].

Variation in genomic DNA is expressed phenotypically in differences in pathogenicity, speed of kill, the production of viral occlusion bodies (OBs), and even the mode of transmission of the pathogen to other susceptible hosts (horizontal transmission) or to the offspring (vertical transmission) [5,6,7,8,9]. These traits are clearly important for virus transmission because they determine the likelihood of the host acquiring an infection [4].

Genotypic and phenotypic diversity have been widely reported for baculovirus isolates from naturally-infected insects. This has involved the characterization of different geographical isolates of the same virus species [10] and also the finding that individual isolates frequently comprise a mixture of genotypes [7,11,12]. In fact, field isolates of baculoviruses generally consist of a number of genotypic variants, which are easily distinguished by restriction endonuclease (REN) analysis [13,14]. This high degree of genetic variability was first described for Autographa californica multiple nucleopolyhedrovirus (AcMNPV) [15] and then for a variety of additional species, including Panolis flammea NPV [16], Spodoptera exigua MNPV [17], Spodoptera frugiperda MNPV [18] and Chrysodeixis chalcites NPV [19], among others.

Diversity within baculoviruses is also important in the development of these viruses as the active ingredients of biological insecticides. Artificial mixtures of selected genotypes have been developed for effective control of different insect pests. These mixtures had useful insecticidal characteristics such as high pathogenicity, virulence, and OB production [20,21,22]. In several cases, the mixtures had improved insecticidal properties over those of the component genotypes or the original field isolates from which they were obtained [19,23]. Genetic diversity in the virus population can also be a valuable tool to avoid the development or overcome resistance in the pest population. For example, multiple resistance mechanisms have been identified in *Cydia pomonella* following extensive use of granulovirus-based insecticides [24,25]. The use of different CpGV genotypes for pest control and insect resistance monitoring are important as individual CpGV variants may fail to control pest populations in certain orchards, which require the use genetically-distinct virus variants [25].

The soybean looper, *Chrysodeixis includens* (Lepidoptera: Noctuidae), is an important agricultural pest that occurs from the northern United States to southern South America. The larvae feed on a range of crop plants, including soybeans, beans, cotton, sunflower, tomato, and potato [26]. This pest is naturally infected by Chrysodeixis includens nucleopolyhedrovirus (ChinNPV), a single-nucleocapsid NPV that is classified as a group II alphabaculovirus [27,28,29]. This highly virulent virus could be a promising biological insecticide for control of *C. includens* [27], particularly given that resistance to chemical insecticides in this pest has become an issue of major concern in some regions [30,31,32,33]. As a result, attention has shifted toward alternative control measures against *C. includens*, including the characterization of different ChinNPV isolates [27,34], with the aim of obtaining novel and genetically diverse variants that may be employed as the basis for an effective biological insecticide.

In this study, we aimed to evaluate the genetic variability of ChinNPV variants present in natural isolates of this virus and their contribution to virus biological activity. We also determined the biological properties of a selection of genotypes that could contribute to the insecticidal properties of the natural field isolates. We conclude that a selection of these variants could constitute the main ingredient of a biological insecticide targeted at this pest.

## 2. Materials and Methods

### 2.1. Insects, Cells, and Viruses

A laboratory colony of *C. includens* was established from larvae collected in soya fields of Tamaulipas, Mexico and maintained under controlled conditions at 25 ± 1 °C, 75% relative humidity (RH) and 16 h light: 8 h dark photoperiod. Larvae were fed a wheatgerm-based semisynthetic diet [35]. HighFive cells from *Trichoplusia ni* (ThermoFisher Scientific, Waltham, MA, USA) were maintained in TNM-FH medium (Gibco, Life technologies Ltd, Inchinnan, Renfrew, UK) with 10% fetal bovine serum (Gibco) at 28 °C.

Isolates of Chrysodeixis includens nucleopolyhedrovirus (ChinNPV) used in this study were obtained from individual *C. includens* larvae that died showing the typical signs of nucleopolyhedrovirus infection. Diseased larvae were collected in 2014 from soya fields in Tamaulipas in north-eastern Mexico, during studies on another soya pest, *Anticarsia gemmatalis* [36]. A total of 105 larvae of *C. includens* were collected in the experimental field station of “Las Huastecas” located in Tamaulipas state, Mexico. Twenty-one of these larvae, representing 20% of the collected larvae, died of polyhedrosis. To purify viral OBs, each virus-killed larva was filtered through muslin and centrifuged with 0.1% SDS several times to eliminate insect debris. The resulting pellets were washed in distilled water and finally resuspended in milli-Q water. OB concentrations were determined using an improved hemocytometer (Hawksley Ltd., Lancing, UK) under phase-contrast microscopy. Purified OBs were stored at 4 °C until required.

### 2.2. Viral DNA Extraction and Restriction Endonuclease Analysis

Virions were released from OBs by incubating 100 μL of OB suspension (10^9^ OBs/mL) with 100 μL of 0.5 M Na_2_CO_3_, 50 μL 10% SDS and 250 μL distilled water at 60 °C during 10 min. The suspension was centrifuged at 6000× *g* for 5 min and the supernatant containing the virions was transferred to a new 1.5 mL microcentrifuge tube and incubated for 45 min at 50 °C with 25 μL proteinase K (20 mg/mL). Viral DNA was then separated from proteins twice with an equal volume of phenol and once with an equal volume of chloroform and precipitated from the aqueous phase using 2.5 volumes of ice-cold absolute ethanol for 10 min at 12,000× *g*. Pelleted DNA was washed twice with 70% ice-cold ethanol and resuspended in 50 μL 0.1× TE buffer (10 mM Tris, 1 mM EDTA pH 8.0). Final DNA concentration was assessed using a Nanodrop One (ThermoFisher Scientific) spectrophotometer.

For restriction endonuclease (REN) analysis, 2 μg of viral DNA of each isolate were digested with BamHI, BglII, EcoRI, HindIII, and PstI (NEB Ltd., Hitchin, UK) for 4 h at 37 °C. The reactions were stopped by mixing with 4 μL of Gel Loading Dye buffer solution (6×, NEB Ltd., UK). Fragments were then separated by electrophoresis in 1% agarose gel immersed in 1× TAE buffer (40 mM Tris, 20 mM acetic acid, 1 mM EDTA pH 8.0) running at 18 V for 15 h. DNA was stained with ethidium bromide and visualized on a UV transilluminator.

### 2.3. Construction of the ChinNPV-Mex1 Mixture and Its Biological Characterization

A sample of 10^8^ OBs of each isolate was obtained from virus-killed field-collected larvae. These samples were used to prepare an equimolar mixture, in terms of OBs, of the nine isolates that we named the ChinNPV-Mex1 mixture.

Initially, to examine the host range of this virus, the ChinNPV-Mex1 mixture was used to inoculate eleven pestiferous lepidopteran species that we had in culture in the UPNA Insectary in Pamplona, Spain, namely: *Anticarsia gemmatalis*, *Chrysodeixis chalcites*, *Chrysodeixis includens*, *Helicoverpa armigera*, *Lobesia botrana*, *Mamestra brassicae*, *Spodoptera eridania*, *Spodoptera exigua*, *Spodoptera frugiperda*, *Spodoptera littoralis* and *Trichoplusia ni*. Two high concentrations of inoculum were chosen, 5 × 10^6^ and 5 × 10^8^ OBs/mL in order to ensure virus-induced mortality in the susceptible species. Inocula were administered to newly-molted second instars of each species by the droplet feeding method [37]. Groups of 24 larvae were inoculated with each concentration. Larvae that drank the OB suspensions within 10 min were individualized in 24-well plates with a piece of semi-synthetic diet. Control larvae drank a solution of sucrose and food dye containing no OBs. Mortality was recorded every 24 h until death or pupation. Results were classified as “non-permissive” (N.P.), or pathogenic in a range from 1+ to 4+, from less pathogenic to highly pathogenic, according to the prevalence of mortality. For susceptible species that experienced mortality in the previous test, 24 newly-molted fourth instars were allowed to drink a suspension of 10^8^ OB/mL of the ChinNPV-Mex1 mixture of isolates. Larvae were then individualized in 24-well plates with semi-synthetic diet. Larvae that died of polyhedrosis were recovered. OBs were purified and DNA was extracted and submitted to REN analyses, as described in Section 2.2, to check the identity of the virus that caused death.

For the biological characterization of the ChinNPV-Mex1 mixture, pathogenicity expressed as 50% lethal concentration (LC_50_), was determined by the droplet feeding method [37]. Newly-molted second instars of *C. includens* were allowed to drink OB suspensions containing 100 mg/mL sucrose and 0.05 mg/mL Fluorella Blue food dye and one of the following five OB concentrations: 1.2 × 10^3^, 6.2 × 10^3^, 3.1 × 10^4^, 1.6 × 10^5^, and 7.8 × 10^5^ OBs/mL, which were expected to cause between 10% and 90% mortality according to results obtained in preliminary tests. Groups of 24 larvae were inoculated with each OB concentration. Those larvae that drank the viral suspension within 10 min were transferred individually to 24-well plates with a piece of semi-synthetic diet. Control larvae drank a solution of sucrose and food dye containing no OBs. Larvae were incubated at 25 ± 1 °C and 75% relative humidity. Virus mortality was recorded every 24 h until larvae had died or pupated. The experiment was performed on three occasions using different batches of insects. Concentration-mortality data were subjected to Probit analysis using the POLO-PC program [38].

The median time to death (MTD) was determined in *C. includens* second instars that were allowed to consume an LC_90_ of ChinNPV-Mex1 OBs, previously determined as 2.3 × 10^6^ OBs/mL. Groups of 24 larvae were inoculated by droplet feeding method, individualized on semi-synthetic diet as described in the LC_50_ bioassay, and were incubated at 25 ± 1 °C. Virus mortality was recorded at 8 h intervals until death or pupation. Control larvae were treated identically but did not consume OBs. The experiment was performed on three occasions using different batches of insects. Before performing a statistical analysis, the results were compared with the available survival time distributions, using the Akaike Information Criterion (AIC) in order to choose the most suitable distribution in R [39]. Data were subsequently analyzed by Kaplan–Meier distribution using SPSS v25.0 [40]

OB production was determined in *C. includens* fifth instars inoculated with an LC_99_ of ChinNPV-Mex1 (10^8^ OBs/mL) by the droplet feeding method. The experiment was performed three times using different batches of insects. Each dying larva was collected in a 1.5 mL microcentrifuge tube to prevent OB loss due to liquefaction of the larva and homogenized in a total volume of 1 mL with distilled water. OBs from 20 randomly-selected larvae per treatment were counted in a Neubauer hemocytometer and each sample was counted three times. As results could not be normalized by transformation, they were subjected to Kruskal–Wallis test using SPSS v25.0 software [40].

### 2.4. Genotypic Characterization and Mortality Response

For genotypic characterization, individual genotypes were isolated from the ChinNPV-Mex1 mixture by plaque purification [41]. Briefly, fifth instar larvae of *C. includens* were inoculated with 10^8^ OBs/mL of ChinNPV-Mex1 by the droplet feeding method. Hemolymph was extracted from 24 larvae by bleeding at 48 h post-infection (p.i.) and immediately frozen. The original sample of hemolymph was diluted in TNM-FH medium, filtered through a 0.45 μm filter and used for plaque purification. After 10 days p.i., wells containing clearly separated plaques were selected for plaque picking. Individual plaques were collected with a sterile Pasteur pipette and diluted in 300 μL of TNM-FH medium. Individual clones were injected intrahaemocelically in *C. includens* fifth instars that were then individualized in a plastic cup with semi-synthetic diet and incubated at 25 ± 1 °C and 75% relative humidity until death or pupation. Dead larvae showing signs of polyhedrosis disease were collected. REN analysis of the DNA (extracted as explained in Section 2.2) of the resultant OBs was performed with EcoRI and HindIII, as the combination of those enzymes allowed a clear discrimination of the different genotypes.

As a first approach, a test of pathogenicity involving one OB concentration close to the LC_50_ (10^4^ OBs/mL) was performed for all the genotypic variants isolated by plaque purification, including the ChinNPV-Mex1 mixture as the reference treatment. Groups of 24 second instar larvae were orally inoculated with OBs of each variant following the droplet feeding method. Those larvae that drank the OB suspension within 10 min were transferred individually to 24-well plates with a piece of semi-synthetic diet. Control larvae drank a solution of sucrose and food dye containing no OBs. Larvae were incubated at 25 ± 1 °C and 75% relative humidity. Mortality was recorded every 24 h. Analysis of the percentage of mortality was performed by Kruskal–Wallis test using SPSS v25.0 software.

### 2.5. Genotype Selection and Characterization of Median Time to Death and OB Production

According to the results obtained in the mortality responses to plaque-purified genotypes, the variants were divided into groups depending on the prevalence of mortality. Thus, one genotype was selected from the group of variants that caused low mortality (less than 15%), two from the group of medium mortality (15 to 30%) and two from the group that caused high mortality (31 to 40%). Median time to death and OB production studies were performed as described in Section 2.3. Median time to death (MTD) was estimated using the log-rank test and OB production (OBs/larva) was subjected to Kruskal–Wallis test, as data could not be normalized by transformation.

## 3. Results

### 3.1. Identification by REN of Field ChinNPV Isolates

**Figure 1:**
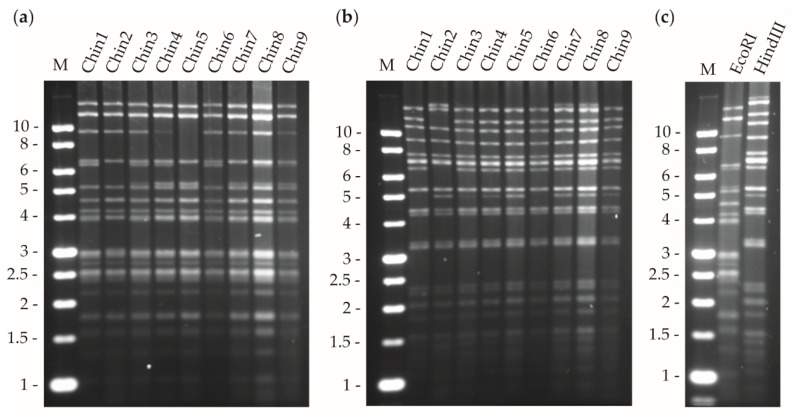
Restriction endonuclease profiles of the genomic DNA of nine different field isolates from virus-killed larvae (**a**) EcoRI, (**b**) HindIII and (**c**) ChinNPV-Mex1 mixture treated with both enzymes. M is the marker and the fragment size is shown in kilobases (Kb) on the left.

DNAs of OBs from 11 virus-killed insects were analyzed by REN to provide evidence for genetic variation. Analyses using EcoRI and HindIII revealed restriction fragment length polymorphisms among isolates, with a total of nine different restriction profiles (Figure 1a,b). The nine different isolates obtained were named Chin1 to Chin9. Two isolates, Chin5 and Chin6, were the genotypes with higher frequency that appeared twice. The REN profile of the ChinNPV-Mex1 mixture corresponded to that of the Chin9 isolate (Figure 1c).

### 3.2. Biological and Genotypic Characterization of ChinNPV-Mex1

The host range study of the ChinNPV-Mex1 mixture showed that this virus was pathogenic to *C. includens*, *C. chalcites* and *T. ni*, all species from the Plusiinae subfamily. The other species tested were found to be non-permissive for this virus (Table 1). REN analysis of genomic DNA of OBs obtained from fourth instar larvae of *C. includens*, *C. chalcites,* and *T. ni* confirmed that ChinNPV was able to cause a productive infection in these insect species.

The pathogenicity of the ChinNPV-Mex1 mixture, expressed as LC_50_, was 6.5 × 10^4^ OBs/mL. Median time to death, analyzed with the log-normal model (selected as the best model for this analysis following a comparison of distribution-dependent AIC values), was estimated at a median value of 125 h post inoculation (hpi) in *C. includens* second instars. The OB production value was 2.2 × 10^9^ OBs/larva in fifth instar larvae (Table 2). None of the control larvae died from polyhedrosis disease.

**Figure 2:**
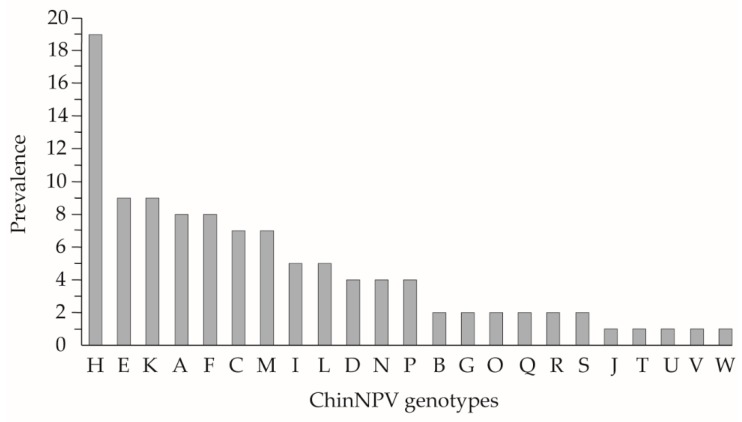
Prevalence of ChinNPV genotypes. Prevalence was calculated as the number of times each genotype was observed in 23 different restriction profiles from 106 clones isolated by plaque assay.

Of the 185 virus-positive plaque picks, only 106 clones caused larval mortality by polyhedrosis following injection in *C. includens* fourth instars. Of the clones amplified in larvae, 23 different genotypic variants were obtained and were named ChinNPV-A to ChinNPV-W. Differences in their genomic restriction profiles were determined following digestion with EcoRI and HindIII. The 106 clones were then classified according to the prevalence of each of the 23 observed restriction profiles. The most prevalent genotype was ChinNPV-H with 19 clones obtained (representing 18% of the plaque-purified variants), whereas genotypic variants ChinNPV-J, T, U, V, and W were only observed in single clones (Figure 2).

### 3.3. Biological Characterization of ChinNPV Genotypes

**Figure 3:**
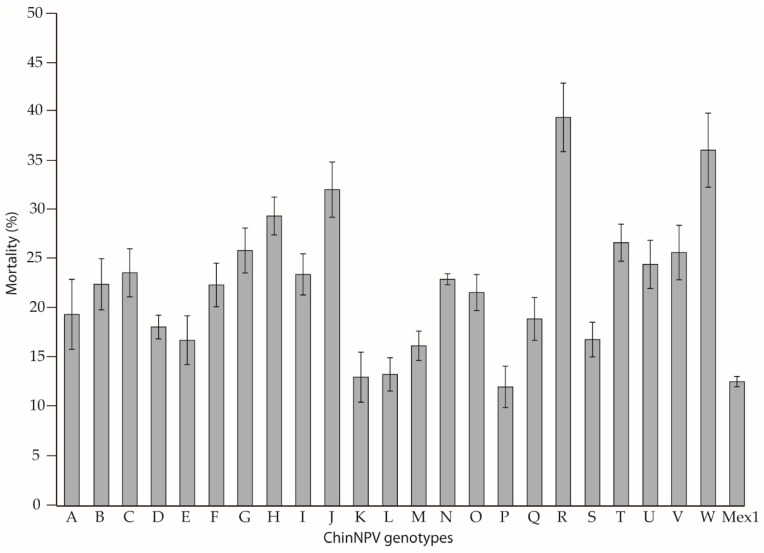
Percentage of mortality for each of the ChinNPV genotypic variants (A–W) and the ChinNPV-Mex1 mixture in *C. includens* second instars inoculated with 10^4^ OBs/mL. Error bars indicate the standard error.

Insect bioassays, performed using a single concentration of inoculum, indicated that the most pathogenic genotype was ChinNPV-R, which resulted in 39.5 ± 7.0% mortality (Figure 3). In addition, statistical analysis revealed that ChinNPV-R was significantly different from the rest of the genotypes (Kruskal-Wallis χ^2^ = 1170, *p* < 0.005; Table A1).

### 3.4. Virulence and OB Production of the Selected Genotypes

**Figure 4:**
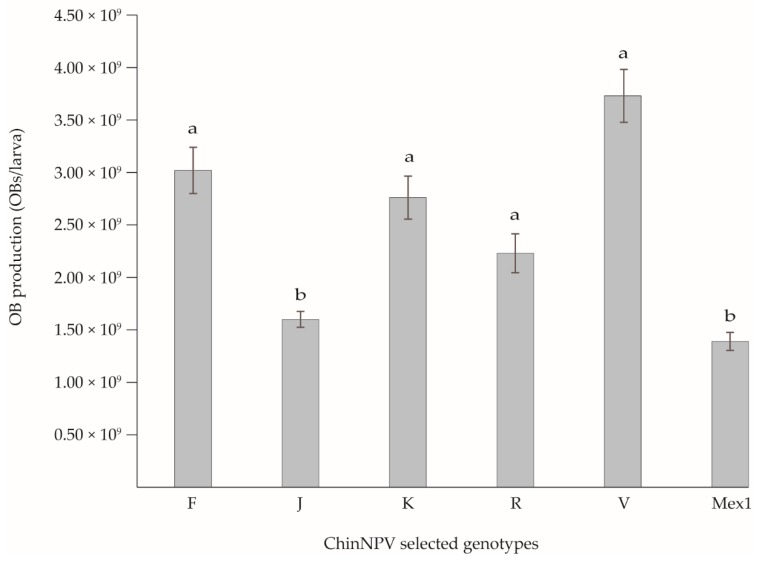
Mean OB production values of ChinNPV-Mex1 and selected genotypic variants in *C. includens* fifth instars. Error bars indicate the standard error. Columns headed by identical letters did not differ significantly (Kruskal-Wallis, *p* > 0.05).

The selected genotypes (ChinNPV-F, J, K, R, and V) did not differ significantly in MTD values and none of them differed significantly from the ChinNPV-Mex1 mixture (Log Rank χ^2^ = 1.096, df = 5, *p* > 0.05; Table 3). OB production estimated in *C. includens* fifth instars showed significant differences, with variants ChinNPV-F, K, R, and V as the most productive genotypes that produced between 2.2 × 10^9^ and 3.7 × 10^9^ OBs/larva depending on variant. This group of variants differed from genotype ChinNPV-J and ChinNPV-Mex1 that produced significantly fewer OBs/larva (Kruskal-Wallis χ^2^ = 19.586, *p* < 0.001, Figure 4).

## 4. Discussion

The genetic variability within field isolates of ChinNPV and the insecticidal properties of their genotypes were examined with the objective of selecting variants with potential for bioinsecticide development. From the eleven corpses obtained from field-infected larvae, nine different REN profiles were obtained, indicating a high variability in natural isolates of this virus under enzootic conditions, given that only 21 larvae died from lethal polyhedrosis out of 105 field-collected larvae (20% mortality). This may be an underestimate of the diversity present in the natural ChinNPV population due to the low number of samples and the omission of non-lethal NPV infection in the present study. The observed variability contrasts with results obtained in the closely-related species *C. chalcites*, a pest of banana crops in the Canary Islands, in which with the natural prevalence of virus-induced mortality of 2.3% resulted in just four different isolates out of 103 infected larvae analyzed from a total of 4438 collected insects [42]. In addition, geographically separated isolates usually show genetic differences, as reported by Alexandre et al. [27], who identified five different isolates of ChinNPV from seven infected *C. includens* larvae obtained at different times and locations in Brazil. These results support the idea that isolates collected from distinct locations tend to differ genetically [43]. In contrast, the ChinNPV used in this study had a behavior and prevalence in field conditions that differed markedly from those described previously. Genetic variability has been proposed as an important tool through which baculoviruses can adapt to changing environmental conditions, and maintaining this genetic variability could even provide a means by which the viruses could adapt to alternative host species [16,44,45]. Nevertheless, the host range of ChinNPV tested in this study was limited to closely-related species in the Plusiinae subfamily, which reflects the high host specificity of most baculoviruses [46]. Consequently, due to this narrow host range, genetic diversity in ChinNPV may not be especially relevant to infection of different host species because although *C. includens* populations naturally coexist with *T. ni* in the soya crop, the virus was not highly pathogenic to this alternative host (Table 1).

The high biological activity of the ChinNPV-Mex1 mixture means that this mixture could be a promising active ingredient for a baculovirus-based insecticide, as the biological activity of this mixture was similar to that of other viruses that are already registered as insecticidal products [21,22,47]. The LC_50_ value of 6.5 × 10^4^ OBs/mL and a MTD of 125 h in *C. includens* second instars suggest that this mixture may be significantly more active than the first registered ChinNPV-based product, Chrysogen, from Brazil, which has an LC_50_ of 1.4 × 10^5^ OBs/mL in *C. includens* neonates [48].

The number of genotypic variants obtained from the ChinNPV-Mex1 mixture was similar to the variability found in other NPVs like Panolis flammea NPV and Helicoverpa zea NPV [49,50]. Although this level of variability has been reported for other viruses, the context changes when the comparison shifts to a more related virus, such as C. chalcites NPV, in which eight genotypic variants were isolated from a single wild-type isolate using the same methodology as used here [19]. For the ChinNPV, 23 genotypic variants were identified from a mixture of nine different wild-type isolates collected from the same area of soya fields at a single time. It is notable however, that this variability was present in a small sample (11 larvae) collected from the same crop at a single moment in time. Although we do not know how the genotypic variants were originally distributed across the eleven infected insects (as the ChinNPV-Mex1 mixture was used as inoculum for the plaque purification), this is still an elevated number of genotypic variants. The fact that *C. includens* larvae infected with ChinNPV and *A. gemmatalis* larvae infected with AgMNPV were collected at the same crop field and time, and that both viruses show high genetic variation [36], indicates that genetically-complex natural NPV populations can coexist in field crop conditions. Indeed, the particular conditions of the crop or field may constitute a source of viral diversity. For instance, the soya crop itself may influence the genetic composition of baculovirus field isolates, as already described elsewhere [51].

For initial characterization of the phenotypic characteristics of variants, a single concentration bioassay was performed, in which the ChinNPV-R variant was the most pathogenic genotype in terms of induced mortality. This was not one of the most prevalent of the plaque purified variants, a finding which supports the idea that in vitro cloning may not accurately reflect the genotypic variation in wild type NPV isolates and may favor genotypes better adapted to cellular culture conditions [7]. Other techniques for studying the genetic structure of NPVs have been developed [52,53], but only in vitro cloning allows the isolation and subsequent characterization of the individual genetic variants. The correlation between genotype prevalence and pathogenicity is however unpredictable. For example, the most prevalent genotypic variant may not be the most pathogenic [18,19,36], whereas other studies report the contrary [23,54,55]. Interestingly, all genotypes induced higher or similar mortalities in experimental insects as the ChinNPV-Mex1 mixture, indicating that genotypic interactions within the artificial mixture may diminish its overall pathogenicity. However, although key to understanding the genetic structure of viral populations, the outcomes of such interactions are usually complex and difficult to predict [4]. Variation in the content of occlusion derived virions within OBs was not evaluated and may have contributed to the observed differences in the pathogenicity of ChinNPV genotypic variants. However, we considered the OB as the infection unit of interest as, under natural conditions, the likelihood of establishing a lethal infection is dependent on the number of OBs that insects consume when feeding on contaminated foliage.

All ChinNPV genotypes were classified into one of three groups (high, medium, and low mortality), according to their capacity to infect and kill larvae. Five genotypes were selected ChinNPV-F, J, K, R, and V, as a representative sample of all the genotypes present in the natural virus population. Speed-of-kill studies demonstrated no marked differences among the five selected variants, none of which was faster-killing than the ChinNPV-Mex1 mixture. In contrast, OB production of ChinNPV-F, K, R, and V variants was higher than the ChinNPV-Mex1 mixture. Taking into account all these results, the genotypes ChinNPV-F, J, R, and V could be candidates for a new bioinsecticide for control of *C. includens*. ChinNPV-J induced high mortality rate but had low OB production, ChinNPV-F and V induced moderate mortality but were both highly productive, and ChinNPV-R had suitable insecticidal characteristics across all the properties that we studied. Nevertheless, due to the fact that ChinNPV-Mex1 showed lower mortality and OB production than most of the individual genotypes, it seems that genotype interactions may result in a reduction in the biological activity of the ChinNPV population. Therefore, the unpredictable nature of virus interactions makes it necessary to test the possible combinations of these genotypes for the design of the final active ingredient of a potential bioinsecticide.

Our results indicate that there is high genotypic variability within the natural population of ChinNPV isolates from north-eastern Mexico. The study of the biological properties of those variants did not explain the reason for the persistence of the high diversity, as the individual genotypes had similar or more active insecticidal characteristics than a mixed-isolate preparation (ChinNPV-Mex1). Most NPV infections involve mixtures of genotypes and the analysis of individual clonal genotypes is unlikely to provide ecologically useful information. In addition, the genotypic heterogeneity seen in natural viral populations facilitates virus survival during stochastic changes in environmental conditions [4]. Although studies are required on how variant interactions may affect phenotypic traits, insecticidal characteristics of the ChinNPV-F, J, R, and V variants appear promising for the development of the active ingredient for a new baculovirus-based insecticide to control *C. includens*.

## 5. Conclusions

The study of the genetic variability in ChinNPV samples collected in a soya field revealed high natural diversity. Nine different isolates were obtained from eleven virus-killed larvae and 23 genetic variants were obtained by plaque purification. Among the variants, ChinNPV-F, J, R, and V were identified as having useful characteristics for the development of a ChinNPV-based insecticide for control of *C. includens*, in terms of pathogenicity, speed-of-kill, and OB production/larva. The results of these studies did not explain the persistence of high diversity in the pathogen population, which may reflect the outcome of variant interactions within infected insects.

## Figures and Tables

**Table 1:** Host range for ChinNPV in second instars of different lepidopteran species.

Species	5 × 10^6^ OBs/mL	5 × 10^8^ OBs/mL
*Anticarsia gemmatalis*	N.P.	N.P.
*Chrysodeixis chalcites*	++	+++
*Chrysodeixis includens*	++++	++++
*Helicoverpa armigera*	N.P.	N.P.
*Lobesia botrana*	N.P.	N.P.
*Mamestra brassicae*	N.P.	N.P.
*Spodoptera eridania*	N.P.	N.P.
*Spodoptera exigua*	N.P.	N.P.
*Spodoptera frugiperda*	N.P.	N.P.
*Spodoptera littoralis*	N.P.	N.P.
*Trichoplusia ni*	+	++++

N.P: Non-permissive. Percentage of mortality: + (1 to 25%), ++ (26 to 50%), +++ (51 to 75%), ++++ (76 to 100%).

**Table 2:** Results of Probit analysis used to estimate median lethal concentration (LC_50_) and median time to death (MTD) values for ChinNPV-Mex1 in *C. includens* second instars and OB production values for ChinNPV-Mex1 in *C. includens* fifth instars.

Virus	LC_50_ * (OBs/mL)	95% Confidence Limits		MTD (h)	95% Confidence Limits		OB Production (OBs/larva)	95% Confidence Limits	
		Low	High		Low	High		Low	High
**ChinNPV-Mex1**	6.5 × 10^4^	2.4 × 10^4^	2.6 × 10^5^	125.0	121.8	128.2	2.2 × 10^9^	1.2 × 10^9^	3.1 × 10^9^

* LC_50_ value estimated from a regression with slope (±SE) 0.826 ± 0.120 and intercept (±SE) −3.981 ± 0.559 (χ^2^ goodness-of-fit test = 3.5641, d.f. = 3, heterogeneity = 1.1880).

**Table 3:** MTD values of genotypic variants estimated in *C. includens* second instars.

Variant	MTD (h)	95% Confidence Limits	
		Low	High
ChinNPV-F	125	120.3	129.7
ChinNPV-J	125	120.4	129.6
ChinNPV-K	119	115.5	122.5
ChinNPV-R	125	120.4	129.6
ChinNPV-V	119	115.7	122.3
ChinNPV-Mex1	125	121.8	128.2

## Appendix A

**Table A1:** Homogeneous subset based on mortality following inoculation with ChinNPV genotypic variants.

Variants	1	2	3	4	5	6	7	8	9	10	11
P	290.080										
A		546.503									
L		679.357	679.357								
K		811.045	811.045								
M			821.033	821.033							
E				948.565	948.565						
S				983.034	983.034						
D				983.703	983.703						
Q					1035.547						
V						1267.990					
O						1282.400	1282.400				
B						1304.449	1304.449	1304.449			
I						1392.007	1392.007	1392.007			
N						1413.618	1413.618	1413.618			
U						1449.109	1449.109	1449.109	1449.109		
C							1451.831	1451.831	1451.831		
F							1467.757	1467.757	1467.757		
G								1664.041	1664.041		
T									1669.211		
H									1670.921		
J										2008.000	
W										2181.689	
R											2409.836
Test statistic	0.0 ^1^	3.078	2.236	7.309	5.246	7.014	10.204	11.173	10.290	1.068	0.0 ^1^
Sig.(2 sided test)	0.0	0.215	0.327	0.063	0.155	0.220	0.116	0.083	0.067	0.301	0.0
Adjusted Sig. (2 sided test)	0.0	0.843	0.952	0.311	0.619	0.613	0.334	0.248	0.235	0.984	0.0

Homogeneous subsets are based on asymptotic significances (*p* = 0.05). Each cell shows the sample average rank of Mortality. ^1^ Unable to compute because the subset contains only one sample.
